# Trees as huge flowers and flowers as oversized floral guides: the role of floral color change and retention of old flowers in *Tibouchina pulchra*

**DOI:** 10.3389/fpls.2015.00362

**Published:** 2015-05-22

**Authors:** Vinícius L. G. Brito, Kevin Weynans, Marlies Sazima, Klaus Lunau

**Affiliations:** ^1^Programa de Pós Graduação em Biologia Vegetal, Laboratório de Biossistemática, Department of Plant Biology, Institute of Biology, State University of CampinasCampinas, Brazil; ^2^Instituto de Biologia, Universidade Federal de UberlândiaMinas Gerais, Brazil; ^3^Institut für Sinnesökologie, Heinrich-Heine-Universität DüsseldorfDüsseldorf, Germany; ^4^Institute of Reconstructive Neurobiology, LIFE & BRAIN Center, University of BonnBonn, Germany

**Keywords:** Atlantic rainforest, attractiveness, bumblebee, color preference, flower–pollinator interaction, mass flowering

## Abstract

Floral color changes and retention of old flowers are frequently combined phenomena restricted to the floral guide or single flowers in few-flowered inflorescences. They are thought to increase the attractiveness over long distances and to direct nearby pollinators toward the rewarding flowers. In *Tibouchina pulchra*, a massively flowering tree, the whole flower changes its color during anthesis. On the first day, the flowers are white and on the next 3 days, they change to pink. This creates a new large-scale color pattern in which the white pre-changed flowers contrast against the pink post-changed ones over the entire tree. We describe the spectral characteristics of floral colors of *T. pulchra* and test bumblebees’ response to this color pattern when viewed at different angles (simulating long and short distances). The results indicated the role of different color components in bumblebee attraction and the possible scenario in which this flower color pattern has evolved. We tested bumblebees’ preference for simulated trees with 75% pink and 25% white flowers resembling the color patterns of *T. pulchra*, and trees with green leaves and pink flowers (control) in long-distance approach. We also compared an artificial setting with three pink flowers and one white flower (*T. pulchra* model) against four pink flowers with white floral guides (control) in short-distance approach. Bumblebees spontaneously preferred the simulated *T. pulchra* patterns in both approaches despite similar reward. Moreover, in short distances, pollinator visits to peripheral, non-rewarding flowers occurred only half as frequently in the simulated *T. pulchra* when compared to the control. Thefore, this exceptional floral color change and the retention of old flowers in *T. pulchra* favors the attraction of pollinators over long distances in a deception process while it honestly directs them toward the rewarding flowers at short distances possibly exploring their innate color preferences.

## Introduction

Floral traits and their patterns in time and space are major keys to understanding plant–pollinator interactions and the diversification of angiosperms (Weiss, 1991a; Lunau, 2003; Schiestl and Johnson, 2013). Traits like color, scent, size, and shape mediate these interactions, advertising to the pollinators the amount and quality of resources and influencing their behavior (Schemske and Bradshaw, 1999; Handelman and Kohn, 2014). Particularly, flower color patterns are important to attract pollinators which are visually oriented at long and short distances, and may affect their flower constancy and preferences (Lunau et al., 1996; Chittka et al., 1999).

Flower color changes during anthesis associated with retention of old flowers is a very common and widespread phenomenon in angiosperms. It occurs in at least 33 orders, 78 families, and 253 genera (Weiss and Lamont, 1997; Suzuki and Ohashi, 2014). Previous studies have shown that this phenomenon creates new attractive units that directly influence the movement of pollinators, favoring both the optimization of the foraging behavior and plant reproduction (Delph and Lively, 1989; Weiss, 1991b). In general, there are two non-exclusive concerted hypotheses to explain flower color changes and the retention of old flowers in angiosperms. For pollinators at long distances, the size of the total floral display will be increased and, so will the number of pollinator visits (Gori, 1983; Oberrath and Böhning-Gaese, 1999). For pollinators at short distances, it is thought that their foraging efficiency will be improved, so that the number of superfluous visits decreases (Weiss, 1995), because the floral color change honestly indicates rewarding flowers (Schaefer et al., 2004).

However, if the pollinators can discriminate the colors of new and old flowers at long distances, the effect of the increased floral display would be worthless, because they can learn to associate floral traits with the amount and quality of reward (Lunau et al., 1996; Raine and Chittka, 2007). Without attraction at long distances, the effects of floral color change at short distance could be achieved without the costs of the retention of old flowers because the flower visitors would not need to probe more flowers when their intention is to visit exclusively pre-changed flowers. Therefore, the old flowers should be similar to new flowers at long distances, while the different floral colors should be discriminable to pollinators at short distances. If this is true, the effect of floral color change and retention of old flowers at long distances can be understood as a deception, while at short distances the same phenomena are honestly signaling the reward to the visitors. Such an effect should be more evident in bee-pollinated flowers, because the spatial resolution of insect compound eyes is very poor and bees will recognize much less details of flower color patterns and see a rough color pattern or even a single mixed color dependent on distance (Vorobyev et al., 1997). Moreover, bees use different sets of photoreceptor inputs at long and short distances depending on the visual angle in which the target is viewed (Giurfa et al., 1996, 1997; Dyer, 2006; Dyer et al., 2008). In this sense, different color attributes should also be important in the visual communication between flowers and pollinators in these plants when we consider long and short distances (Casper and La Pine, 1984; Lamont, 1985). Thus, we expect that green contrast (Chittka, 1992) and spectral purity (Lunau et al., 1996), play different roles in the communication at long and short distances between plants retaining old flowers with an altered color and their pollinators.

**FIGURE 1:**
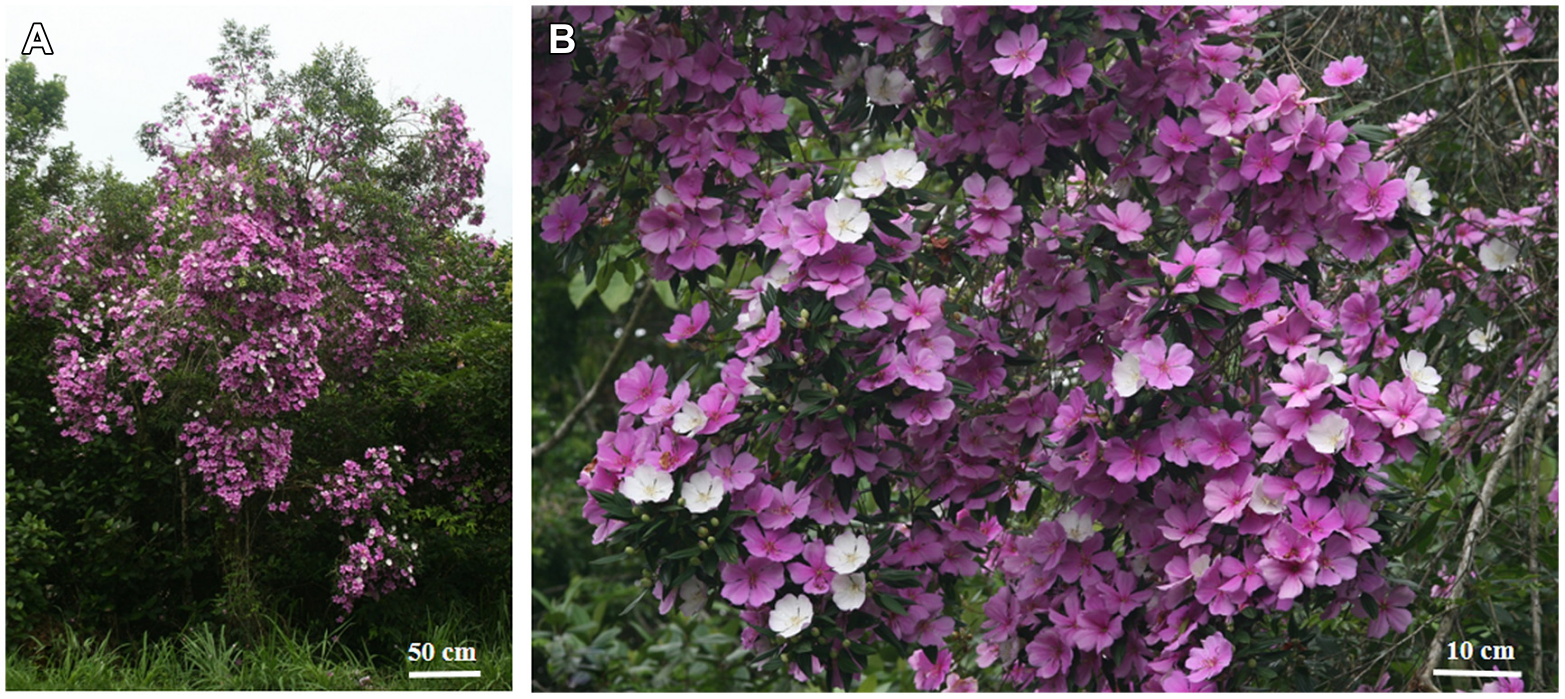
**Floral color change and retention of old flowers in *Tibouchina pulchra*. (A)** A *T. pulchra* tree in flowering peak, covered by old pink flowers, and new white flowers. **(B)** Close-up showing the pink background made by old pink flowers spotted with new white flowers.

Flower color change may be the consequence of a pollination event or be related to the natural senescence of the flower (Gori, 1983, 1989; Pohl et al., 2008). In most of angiosperms, and in several species of Melastomataceae this change occurs just in some small parts of the flowers, e.g., the base of the petals, floral guides, filaments, or ovaries, whereas color change in the entire corolla is more rare (Weiss, 1991b). In *Tibouchina pulchra* Cogn., a common massively flowering tree of the Atlantic Rainforest, the color of the whole flower, including petals, stamens, and style, changes from white on the first day of anthesis, when flowers are rewarding, to pink on the second to the fourth day, when they receive few or no visits (Pereira et al., 2011; Brito and Sazima, 2012). This creates a color pattern that covers the entire crown of the tree, in which the newly opened white flowers are presented in a pink background of old flowers (**Figures 1A,B**). On the other hand, other closely related *Tibouchina* species from the same phylogenetic clade (Michelangeli et al., 2013) change their color from white to red in a very small area at the center of the purple corolla and these flowers are presented in inflorescences scattered in the green foliage. These differences among *T. pulchra* and its congeners highlights this system as a good model to understand the evolutionary meanings of flower color change and retention of old flowers using an experimental design based on a natural condition.

Here we describe *T. pulchra* color patterns considering pollinator’s visual abilities, and add new insights on the phenomenon of floral color change and retention of old flowers in angiosperms. Our main goal is to describe the spectral characteristics of floral colors of *T. pulchra*, to test flower-visiting bumblebees’ response to this color pattern at different visual angles (as a proxy for distance), and to discuss the possible scenario in which this pattern has evolved. We specifically address the following questions: (i) how is the color change viewed when we consider the bee visual system? (ii) is this floral color change discriminable by the bee visual system? (iii) how do the relative spectral purity and the green contrast vary during anthesis? (iv) do these changes in color attributes favor the attraction of bees at long and short distances? (v) do naive bees prefer color patterns produced by *T. pulchra* viewed at long and short distance over the color patterns shown by congeneric species?

## Materials and Methods

### Study Species and Site

*Tibouchina pulchra* is a common hermaphroditic flowering tree that occurs in disturbed sites and secondary forests in the Atlantic Rainforest of Brazil (Leitão-Filho et al., 1993). The large flowers are heterantherous and herkogamous, produce a weak scent and interact with bumblebees able to buzz the poricidal anthers and transfer the pollen to the stigmas of conspecific self-compatible flowers (Pereira et al., 2011; Brito and Sazima, 2012). This pioneer species produces many gravity-dispersed seeds with high germination rates and often quickly colonizes disturbed areas (Zaia and Takaki, 1998).

The data collection of flower colors was made in the summer of 2012 at the Núcleo Picinguaba, Serra do Mar State Park, Ubatuba municipality, located on the northern coast of São Paulo state, Brazil (23°20 S, 44°50 W). The climate is tropical and rainy, with a super-humid season from October to April (Morellato et al., 2000). The mean monthly temperature was 21.2°C and the mean monthly precipitation was 174.7 mm between 2011 and 2013 (CPTEC, 2014).

### Floral Colors

We took normal color and UV photographs of flowers in each of the 4 days of anthesis. Afterward, we excluded the red information provided by normal photography and included the UV information as follows: we split the three color channels of normal photographs (blue, green, and red) and replaced the blue channel by the red channel of UV photography (as the red sensor is UV-sensitive), the green channel by the blue channel of the normal photography and the red channel by the green channel of the normal photography. By this means, we could discern the floral color patterns of new and old flowers perceived by the visual system of a bumblebee. All this procedure employed the sofware Jasc Paint Shop Pro 9. The analysis of photographs in order to visualize bee-subjective colors has recently been advanced by Garcia et al. (2014) using digital images for representation of the spatio-chromatic signal variability. We also measured the spectral reflection of the bases and tips of petals from the first, second, third, and fourth days flowers from 15 trees. These measurements were made using a USB4000 spectrophotometer (Ocean Optics, Inc.) coupled with a deuterium–halogen light source (D2H; World Precision Instruments, Sarasota, FL, USA) able to emit light between 215 and 1700 nm. All the measurements were taken at an angle of 45° to the petal surface and at the same direction relative to the petal (Chittka and Kevan, 2005). We used barium sulfate as white standard and a black film can as black standard for recordings of the spectral reflection (Lunau et al., 2011). We used a standard background of green leaves and a standard daylight illumination (D65, Wyszecki and Stiles, 1982), as well as the spectral sensitivity functions of bumblebee (*Bombus terrestris*) photoreceptors, to calculate the color locus of each measurement using the color hexagon, a model to understand the bee-subjective view of the flowers (Chittka, 1992; Lunau et al., 2011).

To estimate the ability of bumblebees to discriminate flower colors, we performed a multivariate analysis of variance (MANOVA) using position of each flower color measurement (base or tip) and days of anthesis as fixed factors and the values of *x* and *y* axis of the color hexagon as the response variable. We calculated the mean euclidian distance in hexagon units between the new white flowers and old pink flowers, and also among pink flowers of different age. As reference, bumblebees can distinguish correctly by 60% between colors with 0.09 hexagon units of perceptual distance (Dyer, 2006). From these color loci we also calculated the green contrast and the relative spectral purity of the base and tip of flowers from the first to the fourth days using the same color hexagon model (Chittka et al., 1994; Lunau et al., 2011). The green contrast is measured as the distance between the target color locus and the central point of the color hexagon representing the locus of the standard green background (Chittka, 1992). On the other hand, the relative spectral purity is calculated as the proportion between the distance of the color locus from the center of the hexagon and the distance of the corresponding spectral locus representing the maximal spectral purity considering bumblebees’ photoreceptor excitation from the same point (Lunau et al., 1996). We built generalized least-squares models considering the flower’s day of anthesis and the positions of measurement as factors, to explore the differences in these components of floral color. We visually checked the standardized residuals vs. the fitted values plot to conclude for the unnecessity of variance heterogeneity control in such models. The results of this analysis were compared *a posteriori* using the day of anthesis as factor in a pairwise *t*-test with false discovery rate (FDR)-controlling procedures (Benjamini and Hochberg, 1995).

### Bee Preference Experiments

We performed bumblebee preference tests using a Y-maze chamber in the laboratory for long- and short-distance color patterns using artificial paper trees and flowers built with colors simulating the actual colors of *T. pulchra* flowers. Each arm of the Y-maze was 140 cm long and the visual perception angle in each experiment was adjusted by moving the attraction units (artificial trees or flowers) back and forward in each arm (Giurfa et al., 1996). We used two neon tubes (OSRAM L58W/72-965 run with 30 kHz, providing about 2000 lux) above each arm of the Y-maze. As white flowers are kept on the tree during 1 day and pink flowers are kept during 3 days, we defined a color proportion of 25% white and 75% pink in all simulations of the color patterns of *T. pulchra*. All the artificial trees and central flowers used were provided with a 50% sucrose solution droplet at their center to serve as a reward. As control treatment we used a common color pattern that occurs in several congeneric *Tibouchina* species belonging to the same clade in Melastomae tribe phylogeny (clade J – Eartern Brazil, *sensu* Michelangeli et al., 2013) in which the base of the petals is white and the tip of the petals is purple (e. g., *T. heteromalla, T. fothergillae*, *T. clavata*, *T.* cf. *langsdorffiana*). In these species, the flowers change the color just in a very small area in the center, from white to red, and, despite the retention of old flowers, they do not flower so massively as *T. pulchra* and present a number of discrete inflorescences among extensive green leaves. Thus we could test whether bees prefer the color pattern of *T. pulchra* produced by the massive flowering and retention of old post-change flowers to a common pattern produced by flowers with minimal (negligible) color change and no association to massive flowering. When developing the experimental setup we considered three behavioral responses of the bees toward the *Tibouchina* color patterns. Naive bees respond due to their innate preferences, but they lose their naivety as soon as they are rewarded; getting no reward is not regarded as a punishment for the bees since empty flowers are common in nature (Lunau et al., 1996). Experienced bees have learnt differences between rewarding and non-rewarding flowers and are supposed to change their preference accordingly (Spaethe et al., 2001; Niggebrügge and Hempel de Ibarra, 2003). A fundamental study about trained bees’ response to novel color stimuli showed that the bees chose novel colors according to their similarity to the trained color. Only if the tested colors were so different from the trained color that no generalization took place, choice behavior was not affected by the trained color but reflected innate preferences (Gumbert, 2000). Recently it was demonstrated that trained bees show spontaneous color preferences only for disctint color attributes, e.g., color purity, overriding learnt preferences for trained color stimuli, but not for other color attributes, e.g., dominat wavelength (Papiorek et al., 2013; Rohde et al., 2013). Here we assumed that these spontaneous preferences might be important for bees in guiding them to rewarding trees as well as to rewarding flowers irrespective of their experience and the amount of reward.

**FIGURE 2:**
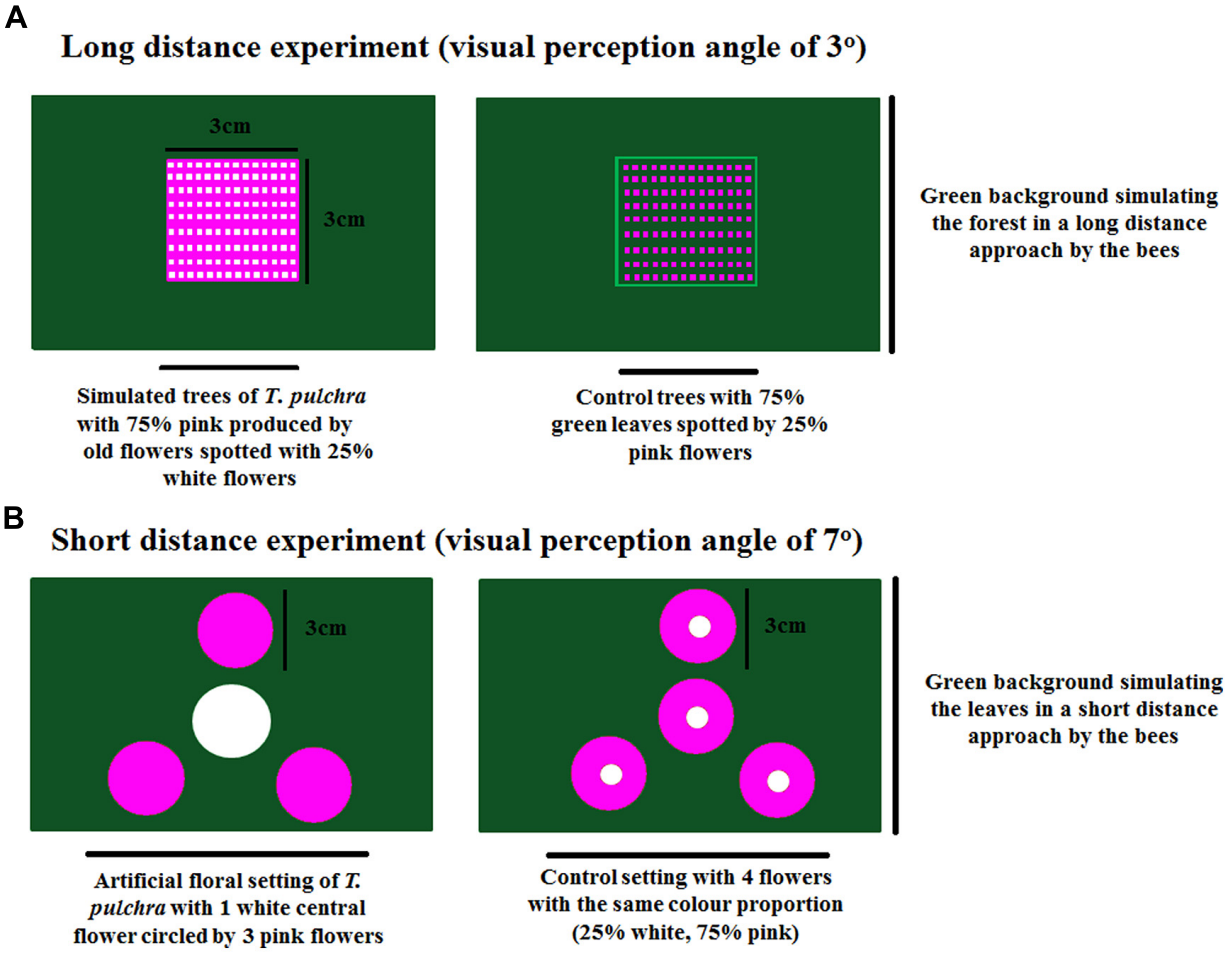
**Experimental settings of long-distance experiment **(A)** and short-distance experiment **(B)** to test the bee preferences in a Y-maze chamber**.

In the long-distance experiment, we used a visual perception angle of 3° (one paper square of 3 cm × 3 cm at 57.28 cm from the decision point). The artificial trees were built with 75% of a pink background representing the old, changed flowers. This pink background was spotted with 25% of white representing the new flowers. The control trees were built with 75% of green leaf background spotted with 25% of pink flowers. In each chamber arm, the trees were presented against a green background simulating the forest where *T. pulchra* occurs (**Figure 2**). With the visual angle of 3° we simulated the crown of a *T. pulchra* tree with 5 m diameter viewed from a distance of 95.5 m. For comparison, a single flower, 5 cm in diameter, can be viewed from a distance of 96 cm under a visual angle of 3°.

We used the same scenario to perform the short-distance experiment with a visual perception angle of 7° (3 cm diameter flowers at 24.52 cm from the decision point). Four *T. pulchra* flowers, one white central flower circled by three pink peripheral flowers, were presented against a green background simulating the green leaves. As control we used four identical flowers with 75% pink on the periphery and 25% white on the center (**Figure 2**). Thus we kept the same color proportion in the simulation of *T. pulchra* flowers and in the control treatment. In this setting, we also tested the number of approaches to peripheral flowers, because only the central flowers were rewarded with sugar solution in both treatments.

We made 12 consecutive trials with 10 naive workers of *Bombus terrestris*, trained once for each trial in the simulation or in the control and in the left or right side of the Y-maze chamber, totalling 120 approaches in long- and short-distance experiments. Therefore we used the treatment, the training simulation and the training side of the chamber as fixed factors and the bee identity as a random factor in generalized linear mixed-effects models with binomial error distribution. All the statistical tests were performed using the R 2.15.0 software using the packages *stats*, *nlme,* and *lme4* (http://www.r-project.org/).

## Results

### Floral Colors

**FIGURE 3:**
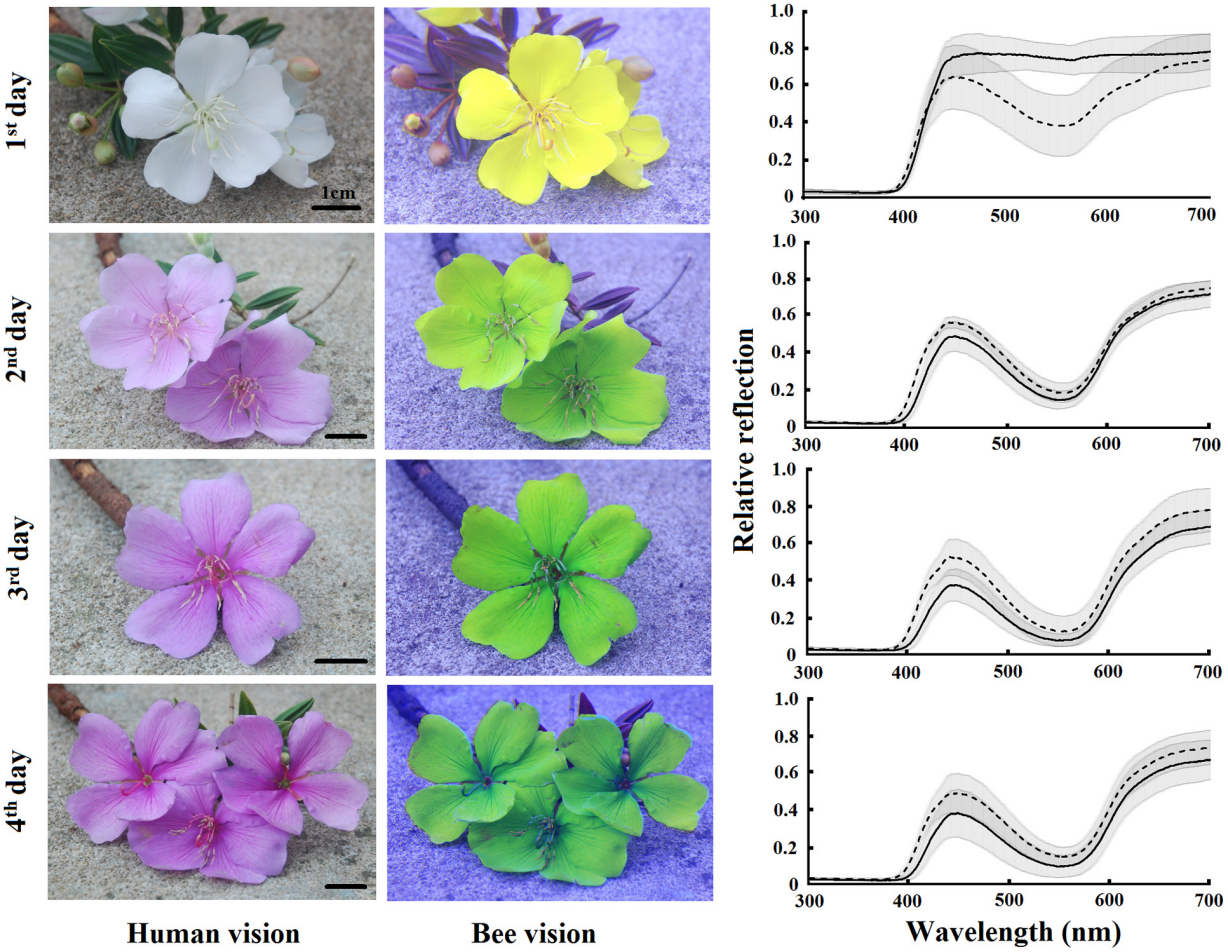
**Color patterns of *T. pulchra* flowers from the first to the fourth days of anthesis.** Photos are shown in conventional photography (red, blue, and green channels) and replacing the blue channel by the red channel of the UV photography (as the red sensor is UV-sensitive), the green channel by the blue channel of the normal photography and the red channel by the green channel of the normal photography in order to reveal the bumblebee color vision perception. Average reflectance curves of the base (solid line) and tip (dotted line) are given for each day of anthesis. Shadow indicates the standard deviation. *N* = 15 individuals.

**FIGURE 4:**
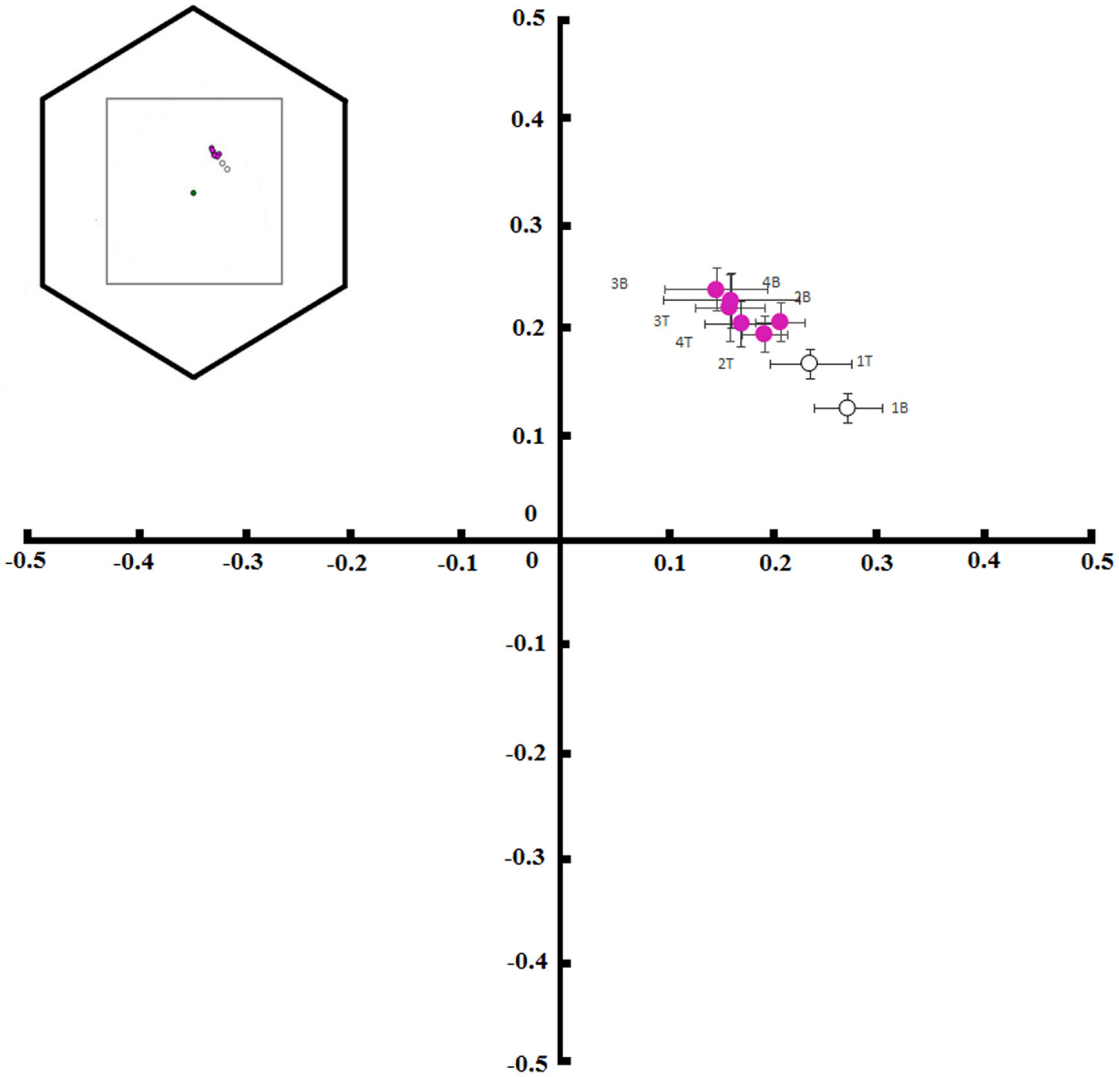
**Color hexagon coordinates showing the locus of base (B) and tip (T) colors of petals from *T. pulchra* flowers.** Color measurements were taken on the first (1), second (2), third (3), and fourth (4) days of anthesis (graph model inspired by Suzuki and Ohashi, 2014).

*Tibouchina pulchra* flowers change their color from white to pink during the 4 days of anthesis (**Figure 3**). When we considered the bee-perceivable color spectrum, there was a decrease in the reflection of green and the flowers become more bee-blue (without UV-reflection; **Figure 3**). This change was abrupt and occured simultaneously in the tip and the base of the petals. Although there was no difference between the color of the petals’ tip and base (MANOVA, *F* = 0.75, *p* = 0.47), the color of flowers of different days was different (MANOVA, *F* = 20.24, *p* < 0.01). The color of these petal parts on the first day occupied a different locus in the color hexagon when compared to the colors on the next days of the same petal parts (**Figure 4**). The mean distance between the new white flowers and old pink flowers was 0.118 ± 0.032 color hexagon units, while the distance among pink flowers on different days was 0.065 ± 0.022 color hexagon units.

**FIGURE 5:**
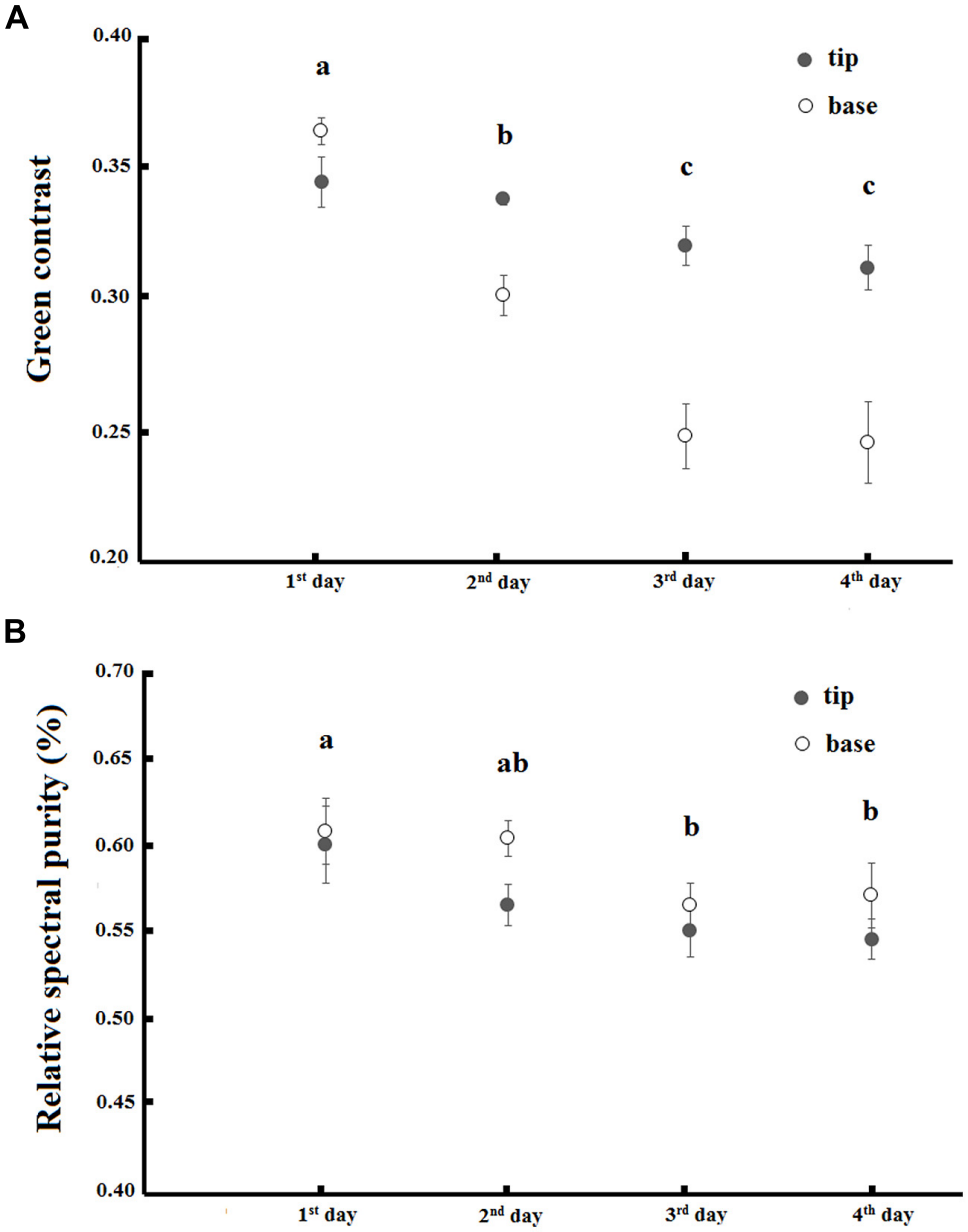
**Green contrast **(A)** and relative spectral purity **(B)** variation along the 4 days of anthesis in base and tip of petals of *T. pulchra* flowers.** Bars indicate SE. Letters indicate significant differences (*p* < 0.05) between the days after pairwise *t*-test with FRD-controlling procedures.

There was an interaction between the day of anthesis and the base and tip of petals when explaining the green contrast component of flower color (*F* = 9.98, *p* < 0.01; **Figure 5A**). The green contrast decreased during anthesis and the change was more pronounced in the base of petals. There was also a decrease in the relative spectral purity of flowers during anthesis (*F* = 4.09, *p* < 0.01), but there was no difference between the base and the tip of petals (ANOVA, *F* = 3.71, *p* > 0.05) and no interaction between these factors occured (ANOVA, *F* = 0.38, *p* > 0.05; **Figure 5B**).

### Bee Preference Experiments

**FIGURE 6:**
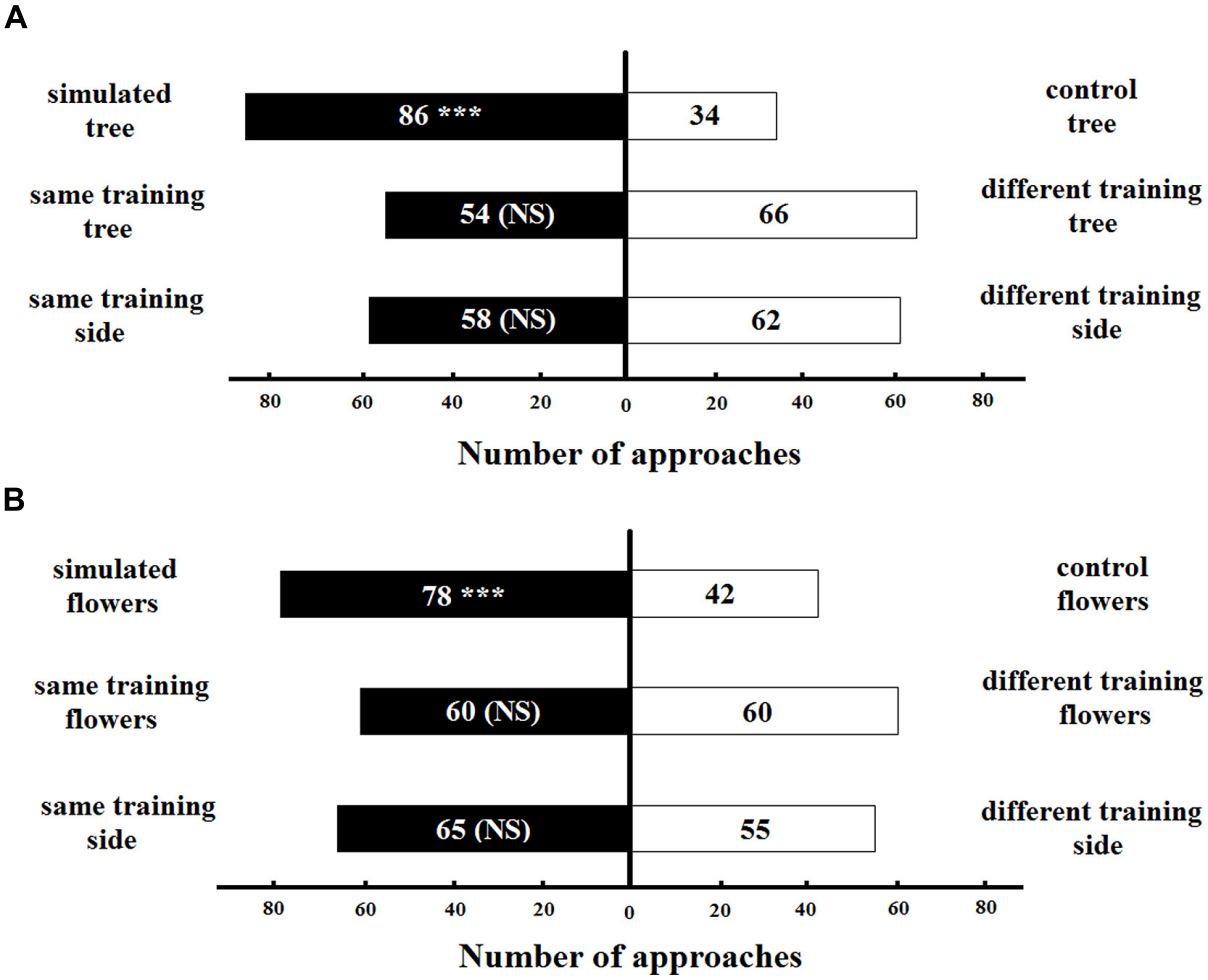
**Number of bee approaches in **(A)** long-distance experiment and **(B)** short-distance experiment simulating color patterns of *T. pulchra*.** ^∗∗∗^ <0.005 significance using a logistic regression model. NS, non-significant.

The naive bumblebees spontaneously preferred the simulated artificial tree with original colors of *T. pulchra* over the tree simulating the congeneric species (control) in the long distance experiments (86 approaches, *z* = 2.50, *p* < 0.05; **Figure 6A**). This result was not influenced by the tree to which the bee was trained (54 approaches to the same training tree, *z* = -1.38, *p* > 0.05) or the side of the Y-maze chamber on which the bee was trained (58 approaches to the same training side, *z* = 1.64, *p* > 0.05). We found a similar pattern for the short-distance experiments: the naive bumblebee preferred the simulation of *T. pulchra* flowers, in which one white flower was circled by three pink flowers, over the congeneric control flowers (78 approaches, *z* = -2.12, *p* < 0.05; **Figure 6B**). This result was not influenced by the training flowers (60 approaches to the same training flowers, *z* = 0.01, *p* > 0.05) or the the training side of the Y maze chamber (65 approaches to the same training side, *z* = 0.33, *p* > 0.05). Moreover, the bees approached only 28 peripheral flowers in the simulated setting of short distance experiments, while they approached 55 peripheral flowers in the control setting (*t-*test, *t* = -2.12, *p* < 0.05).

## Discussion

The floral color change in *T. pulchra* occurs during the 4 days of the anthesis and the whole flower changes its color from white to pink in the human visual system. However, when we consider the bee visual system the change occurs mostly at the base of the petals in the green range of wavelength, which becomes less prevalent in the flower color composition during the anthesis. The bees cannot easily discriminate old flowers from different days of anthesis due to the small perceptual distance between their color loci. However, the new white flowers are distinct from the old pink flowers when we consider the distance of 0.09 hexagon units as a bee discrimination threshold (Dyer, 2006). This result indicates that *T. pulchra* post-changed flowers, when retained at the treetop, create a background different from that of green leaves, covering the whole tree where the new flowers are exposed to pollinators. This pattern should be even more conspicuous for the bees once the production of green leaves decreases during the flowering time in *T. pulchra* and the treetop is covered almost exclusively by the new and old flowers (Brito and Sazima, 2012).

In general, the retention of old flowers has been suggested to be a strategy to attract more pollinators at long distances by increasing the floral display size (Gori, 1983; Weiss, 1991b; Kudo et al., 2007). On the other hand, bees are unable to resolve the distinct parts of the color pattern at long distances, which limits their capacity to discriminate fine color patterns from a uniformly colored area at long distances (Dafni et al., 1997; Hempel de Ibarra et al., 2014). Thus, it should be assumed that at long distances the bees see a single mixed color composed by the colors of the color pattern and their ratio. The results of the long-distance experiment indicate that the bees prefer the *T. pulchra* over the hypothetical ancestral tree. In this sense, it is noteworthy that *T. pulchra* does not display red (or subjective bee black) as a post-change color as many other flowers including other *Tibouchina* species do (Weiss, 1991a; Weiss and Lamont, 1997), because bees have a very limited ability to see red (Lunau and Maier, 1995). Since all colors involved absorb ultraviolet light, white as well as pink is spectrally pure bee-bluegreen, whereas red would appear similar to the green background color to which the eyes of the bees are adapted. The spectral reflectance properties of white and (pale) pink are more similar than that of white and red, and this should increase the overall attractiveness of the mixed colors of the tree in the forest gaps. However, it is still no trivial question whether the progress in attractiveness was mediated by the color mixed of one quarter white and three quarters pink of *T. pulchra* over the hypothetical ancestral color mixed of one quarter pink and three quarters green or the increased display size of a colored target object or even an interaction of these factors.

The short-distance experiment also showed that bees prefer the simulated *T. pulchra* floral composition to the control, in which the same amount of white and pink colors was presented. Moreover, in this experiment the bees made fewer mistakes (approaches to peripheral flowers) in *T. pulchra* simulated flowers, as was foreseen by the short distance hypothesis to explain floral color change (Weiss, 1995). When floral color change is associated with retention of old flowers, the color differences at short distances should be associated with differences in floral resources to encourage pollinator visits (Lamont, 1985). Moreover, such color pattern associated with differences in reward, should be strengthened by the differences in scent between new and old flowers (Pereira et al., 2011). In the nectarless *T. pulchra* flowers the pollen is almost depleted during visits to the new white flowers, and therefore, visits to old pink flowers are rewardless and thus rare or non-existent (Pereira et al., 2011; Brito and Sazima, 2012). When pollinators restrict their visits to newly opened white flowers, they increase their efficiency by getting more pollen per visit, besides promoting pollination and avoiding pollen wastage (Weiss, 1995). Moreover, the color pattern of *T. pulchra* should also favor the movement of bees to longer distances, promoting outcrossing among different trees (Harder and Barrett, 1995; Sun et al., 2005).

The phenomenon of floral color change in plants is closely linked to the pollinators’ ability to learn and associate color with the amount and quality of reward (Lunau et al., 1996; Raine and Chittka, 2007; Pohl et al., 2008). In *T. pulchra*, a species strictly dependent on large bees to set fruits (Brito and Sazima, 2012), the floral color attributes should also be important for the functioning of the strategy of floral color change considering long and short distances. In addition to the low spatial visual resolution, bumblees are unable to distinguish colors at long distances, because they use only the information from green photoreceptors when the visual angles are lower than 2.7° (Dyer et al., 2008). Other bee species might use a deviant critical visual angle, e.g., for the Western honeybee the critical visual angle is 15° (Dyer et al., 2008), which means that a bumblebee is able to detect a *T. pulchra* tree from a distance that is more than five times larger than that of a honeybee. Bumblebees detect stimuli containing both green-receptor-contrast and color contrast at a visual angle of approximately 2.3°, whilst stimuli that contain only color contrast are only detected at a visual angle of 2.7° (Dyer et al., 2008). On the other hand, the respective viewing angles for honeybees amount to 5° and 15°. The maximal detection distance for a *T. pulchra* tree possessing a crown of 5 m diameter for bumblebees via green contrast amounts 125 and 106 m via color contrast, whereas honeybee have to approch to 57 m to detect the tree via green contrast and up to 19 m to detect it via color contrast. Therefore, the calculation of the maximal detection distance for *T. pulchra* trees presumes that bees would detect the grouped flowers better than single flowers. The grouping of flowers into patches, experimentally simulated by three spatially separated disks – similar to the the experimental design in our study – as compared to a single disk improved their detectability by bees and such improvement of detectability should be stronger for bumblebees than for honeybees (Wertlen et al., 2008). Thus, in a long-distance perspective the pattern composed by the new and old flowers in *T. pulchra*, as well as the retention of these flowers in the tree as a whole, provides a large, attractive and deceptive object in the gaps of the forest for the bees. The attractiveness of the whole tree should be given by the high spectral purity values of new and old flowers, which also favors the discrimination of the trees in the green forest background while it does not indicate the differences between new and old flowers. In fact, experienced bumblebees exhibit a preference for spectrally purer colors over trained colors even if the perceptual color distance is small (Rohde et al., 2013). When this stimulus is perceived at long distance and an approach is made, the bee vision changes automatically to a color vision in which all the photoreceptors are used (Giurfa et al., 1996, 1997; Giurfa and Lehrer, 2001; Dyer et al., 2008). In this context, the new rewarding and the old rewardless flowers of *T. pulchra* can be honestly discriminated and dominant wavelength, associated with the differences in the green contrast among flowers from different days, should be the major mediator of the bee attraction process at short distances.

In general, bees have an innate preference for high spectrally pure and contrasting colors and this may explain the color patterns of flower structures in angiosperms (Lunau and Maier, 1995; Lunau et al., 1996). Mostly, the floral guides display large visual contrast against the corolla and higher spectral purity than the corollas. This set is more spectrally pure than the green leaves, creating a unidirectional color pattern of increase in spectral purity that may direct the pollinators to the rewarding sites and reproductive structures of the flower (Lunau, 1996). Floral color change is also present in other *Tibouchina* species, in which this change occurs only in the white base of the petals, while the periphery remains with the same color, often purple (e. g., *T. heteromalla, T. fothergillae*, *T. clavata*, *T.* cf. *langsdorffiana*). This color pattern creates the unidirectional floral color disposition in new flowers that is suspended in the old ones. As this color pattern is very common along the *Tibouchina* Brazilian clade (clade J – Eastern Brazil, *sensu* Michelangeli et al., 2013), we stated that, in *T. pulchra*, the retention of old pink flowers together with massive flowering favored an enlargement of the previous floral guide to the whole periphery of the corolla. In fact, some individuals do present a pink color in the petals’ tip of their new flowers, probably a vestige of a pink corolla. Therefore, *T. pulchra* trees completely covered by flowers function as a huge flower in forest gaps and the new white flowers function as oversized large floral guides, guiding the pollinators to the reward and the reproductive floral structures.

The floral color change of *T. pulchra* is exceptional in multiple aspects. Floral color change in most flowers is restricted to floral guides or small flowers in many-flowered inflorescences (Weiss, 1991b), whereas *T. pulchra* has large flowers which change their color in the entire visually signaling apparatus. In some plants, the floral color change is triggered by pollination (Gori, 1983; Van Doorn, 2002), but in *T. pulchra* color change indicates senescence and is associated with flower duration. Moreover, flowers dominate the visual display of the entire plant of *T. pulchra*, whereas green leaves normally dominate it in other plants. This has enabled us to use *T. pulchra* as a model plant to test experimentally the sustainability of long distance attractiveness hypothesis for the first time. However, it remains open whether the color pattern, the display size or a synergetic effect of both is responsible for this bee preference at long distances, because these parameters were combined in our experimental setup. Future experiments might disentangle which of these parameters have been important for the evolution of flower color change and retention of old flowers. Future studies also might show whether the special floral color change of *T. pulchra* was favored by the scattered distribution of the plants in rare disturbed areas, once it would favor the attraction of the pollinators from very large distances matching the average distances between single trees. Since the visual attention in a complex search task differs between honeybees and bumblebees (Morawetz and Spaethe, 2012), the long distance signaling of flowering *T. pulchra* trees might represent a strategy to selectively address bumblebees, solitary foragers that largely rely on their own experience, instead of mass-recruiting honeybees and stingless bees. Recent studies have highlighted different search strategies in bees depending of environmental consitions and intracolony-communication (Morawetz and Spaethe, 2012; Bukovac et al., 2013); however, it is still unknown whether tropical bumblebees, the most frequent flower-visitors and pollinators, possess a distinct search strategy. In this regard it seems noteworthy that the nectarless flowers of *T. pulchra* are not attractive to honeybees that are not capable of buzzing the flowers for pollen reward. *T. pulchra* thus might benefit from adjusting their visual display for pollinating bumblebees.

In this study, the long-distance experiment demonstrated that naive bumblebees prefer the simulating trees with color change over the control model in which there was no color change or massive flowering. The same preference was found in the short-distance experiment demonstrating that both long- and short-distance hypotheses can explain this phenomenon in angiosperms, as previous studies have shown (Casper and La Pine, 1984; Delph and Lively, 1989; Weiss, 1991b; Weiss and Lamont, 1997; Ida and Kudo, 2003). Furthermore, we suggest that different attributes of floral colors play different roles in long and short distances regarding the attraction and direction of pollinators among flowers. Moreover, because we performed our experiments with naive bumblebees, the results reinforce the idea that floral color change creates color a patterns that increase unidirectionally the attractiveness from the greens leaves to the new flowers. In this sense, floral color change when associated with retention of old flowers, could be favored in angiosperms by exploring innate preferences of bees (Lunau, 1996; Papiorek et al., 2013).

## Conflict of Interest Statement

The reviewer Adrian G. Dyer declares that, despite having collaborated with the author Klaus Lunau, the review process was handled objectively and no conflict of interest exists. The authors declare that the research was conducted in the absence of any commercial or financial relationships that could be construed as a potential conflict of interest.
